# Tracheotomy and Laryngeal Injections in the Throat

**Published:** 1891-04

**Authors:** J. C. Meyers


### TRACHEOTOMY AND LARYNGEAL INJECTIONS IN THE THROAT.

By J. C. Meyers, M. D., V. S.

Diseases of the throat described in every text book, and their frequent appearance in veterinary medicine must necessarily make the practitioner more or less familiar with their characteristics and gradations, therefore it would be, not only superfluous, but also improper, to consume the time by recapitulating the symptoms, causes, and particularly, as in many of the ordinary cases, therapeutic interference, is not wanted. Last fall we had many opportunities to observe the last named form, as at one time the disease threatened to become enzootic. The milder sore throat cases, which were in the majority, suffered mostly from catarrhal pharyngitis, *Angina Pharyngitis.*

Among those affected with laryngo pharyngitis, several either took sick suddenly, or for which medical assistance was postponed until suffocation threatened to cut short existence; thus necessitating the operation of tracheotomy. For this purpose I prefer the trocar, invented by Prof. Hayne, of Vienna, especially when the acuteness of the case indicates a probability, that the tube will not be wanted any longer than a week or ten days, as in croup, purpura hemorrhagica (Blutflecken krankheit) cramp of the larynx, etc. The technical procedure with this trocar requires the least time, there is no loss of substance, and the cannula is not difficult to clean. The caliber, however, is too small to allow such an animal to work with. Prof. Vogel and Prof. Prosch are less favorably impressed by it, the former recommends Thompson's as the most suitable. Besides other objections they claim there is danger in operating with the Hayne instrument in that it is likely to pierce a jugular. I once thought to have met with such an accident, a short account of which may not be out of place.

One evening before sunset summons reached me to come to see a dray horse which just came in from work, whistling and roaring bad enough to suffocate if not relieved soon. Provided with the instrument in question I hurried to the place. Approaching the scene of trouble, I could distinctly hear the described noise notwithstanding the crowd collected around the animal. Drawing nearer I saw a well-nourished black horse, very uneasy, with an anxious look, pumping flanks, and foaming from mouth and nose. Under these circumstances I was obliged to operate without delay. I immediately grasped the trachea with the thumb index, and middle fingers of the left hand, about four inches below the larynx, pushing the sterno maxillary muscles a trifle backwards from their lateral position, giving the tracheotome a passage free from all other tissues; the trachea was then pierced through from left to right in an exact transverse direction, with the instrument held in the right hand, so that the central opening in the tube remained in the middle of the windpipe. The stylet was taken out and the screws, (to make the cannula stationary) adjusted with allowance for some swelling. As the skin is the most sensitive tissue, it is advisable to first make an incision through it with a knife, before the trocar enters. Usually upon withdrawal of the stylet air will follow. But alas! this time both openings discharged as much blood as their lumen would allow. The bystanders knew no better, but that I was bleeding the horse, which they thought was well done, but my enthusiasm was easily controlled; the thought that the opposite jugular might have been struck, appalled me; still I managed to keep calm, walked leisurely around to the other side of the horse, inspected the outlet of the tube and found my supposition erroneous, I gained courage; at the same time the hemorrhage diminished and in 1-2 to 3-4 minutes ceased entirely as also did the blowing and puffing. The cannula was removed, the patient allowed to go to his stall, when he commenced eating hay.

During a fit of coughing he expelled some blood clots mixed with slime; perhaps a false membrane. The owner informed me that on the same afternoon the horse had performed hard work, to which exertion I attributed the formation of a hæmatoma saccatum which the instrument must have penetrated producing the critical symptoms alluded to above. Four days after he was at work again. Now if the more popular operation, with excision of a suitable piece of the trachea is resorted to, the reconvalescence requires more time and as we all know, will not admit of a complete restoration, though it is seldom harmful. Should the nature of the case require an artificial opening for several weeks or longer, particularly if the animal is obliged to work, a tracheotomy tube of a later pattern, consisting of two parts, arranged and attached in such a manner as to attract but little attention is preferable.

Circumstances may compel us to deviate from the custom in the selection of a tube. Tumefaction of the neck at the outset, or the development of the same around the artificial opening, occasionally requires a change of the tube, in which case the operator must use his own judgment.

Recently I was called upon to treat another horse with roaring and difficult respiration. The driver told me that about two month's previous the horse had a similar attack of about thirty minutes duration. Since which time he evinced nothing wrong until now. As there was no swelling detectable around the neck or maxillary region, but repeated convulsive coughing, sweating and a frothy discharge from the nostrils, I presumed it must be "Spasmus Glottidis," an ailment known to me only through literature. Whether my diagnosis was correct or not, the symptoms indicated the operation which was performed, giving relief to the horse and all interested. The Hayne trocar was again chosen, expecting the horse would not need it any longer than a few days. Despite the original symptoms and anamnestic report indicating a spasmodic affection of the larynx I was obliged on the following day, to change my diagnosis to Glottis Oedemia, owing to the phenomena which developed through the night. All went well until the eleventh day when I plugged the openings of the tube, as I thought, sufficiently long to produce wheezing if the air passages were not clear, removed the cannula, cleaned it etc., but in eight or ten minutes after he commenced roaring again. My expectation not being realized, I felt obliged to insert another tube of a larger caliber, which served two weeks longer, when the horse seemed to be all right with the exception of the healing of the wound; whilst this was going on the horse received daily exercise without showing any deficiency. But we were destined to be disappointed, for in the first days hard work, it was found that his respiration was not yet normal. The proposition to send him to pasture for a few months, met with approval, from whence we receive most satisfactory reports.

The active participation taken by some of our most progressive surgeons in the laryngeal operation "Laryngotomy," recorded in the *American Veterinary Review*, leads us to believe that diseases of the throat are attracting more attention than heretofore.

Considering these facts a translation of an article on "Spasmus Glottidis," by Prof. Anancker in *Kochs Encyclopedia der Thierheilkunde*, will undoubtedly be appreciated. It reads as follows :

"Spasms of the glottis, spasmus glottidis consists in tonic contractions of the lateral crico arytenoid, the inferior and superior thyro-arytenoid, and the transverse arytenoid muscles, secondly, also the muscles of the neck, as in asthma, the cause has been sought for in the reflex irritation of the nerves of the larynx, consequently the spasmodic attacks recur at uncertain intervals. As the glottis becomes contracted during glottis spasms it produces a dyspnœa attended with loud sounds during inspiration, therefore it was enumerated as one of the causes of roaring, but Gunther Senior, doubts this, as he did not see it set in upon intentionally irritating the upper laryngeal nerve. That such a spasm can be brought about is not to be questioned, at least, spasmodic cases in horses, have been observed. The spasmodic attacks recur sometimes daily, even while the horse is at work; connected with them are cough and suffocating spells, sometimes restlessness, anxiety, shivering, distended nostrils, rattling in the throat, stretching of the head, staring look, staggering movements and collapse; characteristic is the sudden abatement of the attacks. In the autopsy of a horse Degive found a cyst as large as a hen's egg filled with colloid matter, at the epiglottis, which on account of its position, could not close the glottis, but may have called forth reflex spasmodic contraction, To obviate threatened asphyxia, tracheotomy must be performed; repeated attacks of spasms must be alleviated by hot water inhalations, or a solution of Kali Bromid. as prescribed for asthma, and by tracheal injections of the same, or a solution of cocaine or morphia, toxic symptoms which may take place will disappear at once, upon inhaling several drops of nitrate of amyl (Anacker)."

As to therapeutic treatment. I am not in possession of any specific remedy, with which this manifold complicated ailment can be checked or influenced much. In benign cases, the owner, with the aid of his infallible neighbor, will, with few exceptions, help himself. The malignant or complicated cases must be treated according to circumstances. It would be difficult to give a formula which would hold good for even a few days.

There is, however, a discovery made known which is worth communicating to those who believe that nature tolerates support when rendered in a judicious manner and at the proper time. At a recent meeting of the German naturalists and physicians at Heïdelberg, colleague Dr. Jelkman, of the section for Veterinary Medicine, reported on "Laryngo Pharyngitis of the Horse and its Cure by Laryngeal Injections of Prussic Acid," wherein he says: "Although the results in the treatment of inflamatory affections of the respiratory mucus membrane in coryza, adenitis, scalma, etc., were very unsatisfactory with the remedies employed so far, namely, to cut short the morbid process, fault must not be found with the physiological actions only, but also in the mode of application. As these agents are introduced mainly through the mouth it is impossible for them to come in direct contact with the affected mucus, and thus a local influence is almost excluded."

Local methods of treatment promised much better results, as through the examination of Prof. Dr. Schuetz it was proven that the diseases in question, nearly always in their primary stage, present a limited infectious inflammation of the surface of the mucous membrane. Again, the experiments of Prof. Dr. Dickerhoff, have also shown that the local treatment of the respiratory mucosa is easily accomplished. Therefore if the proper remedy be found the result will be satisfactory, if the local treatment be adopted in time. Jelkman being guided by the above views made numerous experiments, and applied many expedients which were recommended, and others which had not yet been put to test. The first opportunity presented itself in a large transfer stable at Frankfurt, *A. M.*, among horses affected with infectious laryngo-pharyngitis, a scalma enzootic.

Among 100 horses in an inclosure sixty took sick within six weeks, at intervals of one, two and four days. It being mid-summer those patients, six in number, which took sick in the first three days, were isolated, and placed in a large, roomy, well-ventilated hall. The internal treatment with the remedies known as promoters and regulators of morbid slime secretions, etc., was carried out from the first to the seventh day, but seemed to have no visible effect, nor did the externally applied derivants. The consequent laryngeal injections were made as an experiment. By this time the number of patients had increased to twelve. Laryngeal injections were administered to ten of these patients, each two receiving a different compound, for example, one consisted of sol. of alum acet., another of lugole's solution, another contained cocaine hydrochloric, and so on. The remaining two of the twelve were intrusted to physiatrice. This treatment was continued for four days without any apparent improvement, only in those having received the cocaine, it was noticed that the coughing irritation grew less soon after the injection, which, however, in a few hours recurred with the same violence. In those left without treatment the disease remained stationary. Taking into consideration the satisfactory results attained in human medicine by applying anodynes to the suffering tissues in inflammatory affections of the larynx and bronchial mucous membrane, an experiment was made with the following solution: morphia hydrochloric 2.0, aq. amygdalar amar. 200.0, 10 grms. of this solution was injected every morning and evening into the larynx of each of five other patients. The result was astonishing. The temperature which was 39.8 to 40.7 C., fell from the day of the first injection until the fifth, by which time the normal state was reached. The local appearances in the region of the œsophagus and mucous membrane of the larynx diminished in the same degree. The cough became loosened, less painful, the dysphagia disappeared and the appetite returned. The hyperplastic swellings of the maxillary glands also dissolved. Of the twelve horses which took sick first and not treated after this manner, not one had recovered sufficiently to be able to perform the lightest work. While those which took sick eight or ten days later and were treated with aq. amygdalar amer. and morphia, were already at their usual work.

In the last two years Jelkman treated all laryngeal affections in horses with positive and speedy success as long as metastasis of the other organs did not set in. If the local inflammatory process of the mucous membrane was in the early stage of development, the disease could be cut short within two or three days, indeed be entirely cured.

If the disease had advanced further, a four to eight days treatment with injections was necessary. It is remarkable that in 200 patients the hyperplastic swellings of the sub-maxillary glands, diminished after the first injection, and in but three cases a slight imperfect abscess formed. After these results there is no doubt that the prussic acid contained in bitter almond water, with morphia, exercises a specific healing effect on the respiratory mucous membrane. Prof. Bing's established non-fermentation and anti putrefactive qualities of prussic acid supports such an opinion.

In the diseases in question, laryngeal injections are most effective. The head is lifted and stretched forward and the needle inserted in the so-called thyro-cricoid space. The contents of the syringe is then emptied under strong pressure. This pressure is absolutely necessary to the favorable result of the injection, for the diseased surface of the mucous membrane should not only be wetted but at the same time washed off. This washing off cannot be accomplished by the gentle inpouring of the fluid into the larynx. Unfavorable manifestations have never been noticed to follow upon injections being thus carried out. If it is inconvenient to inject twice per day, one injection of 20 grms. will suffice. When the weather is cold it is advisable to warm the fluid before injecting. Abscesses, that may form after injection will heal readily, when lanced in time and cautious antiseptic treatment is pursued.

